# Metformin inhibits the histone methyltransferase CARM1 and attenuates H3 histone methylation during gluconeogenesis

**DOI:** 10.1016/j.jbc.2025.108271

**Published:** 2025-02-06

**Authors:** Sinjini Dhang, Atanu Mondal, Chandrima Das, Siddhartha Roy

**Affiliations:** 1 Structural Biology and Bio-Informatics Division, Council of Scientific & Industrial Research-Indian Institute of Chemical Biology, Kolkata, India; 2 Academy of Scientific and Innovative Research (AcSIR), Ghaziabad, India; 3 Biophysics and Structural Genomics Division, Saha Institute of Nuclear Physics, Kolkata, India; 4 Homi Bhabha National Institute, Mumbai, India

**Keywords:** epigenetics, metformin, CARM1, histone methylation, enzymes, gene expression

## Abstract

Hyperglycemia is a hallmark of metabolic disorders, yet the precise mechanisms linking epigenetic regulation to glucose metabolism remain underexplored. Coactivator-associated arginine methyltransferase 1 (CARM1), a type I histone methyltransferase, promotes transcriptional activation through the methylation of histone H3 at arginine residues H3R17 and H3R26. Here, we identify a novel mechanism by which metformin, a widely prescribed antidiabetic drug, inhibits CARM1 activity. Using biochemical and biophysical assays, we show that metformin binds to the substrate-binding site of CARM1, reducing histone H3 methylation levels in CARM1-overexpressing hepatic cells and liver tissues from metformin-fed mice. This epigenetic modulation suppresses the expression of gluconeogenic enzymes (G6Pase, FBPase, and PCK1), thereby reversing CARM1-induced glycolytic suppression and regulating gluconeogenesis. Importantly, metformin does not alter CARM1 protein levels and its recruitment to gluconeogenic gene promoters but diminishes H3R17me2a marks at these loci. Our findings reveal a previously unrecognized epigenetic mechanism of metformin action, offering new therapeutic insights for hyperglycemia management.

Protein arginine methylation represents a crucial posttranslational modification that plays a fundamental role in regulating chromatin dynamics and gene expression (1, 2). This essential cellular process is catalyzed by protein arginine methyltransferases (PRMTs), a family of enzymes whose dysregulation has been increasingly linked to various pathological conditions. The aberrant expression or activity of PRMTs has emerged as a significant factor in disease progression, particularly in cancer development and metabolic disorders (3, 4, 5, 6). Among the PRMT family members, coactivator-associated arginine methyltransferase 1 (CARM1), also known as PRMT4, has garnered substantial attention due to its diverse physiological roles. Initially characterized as a transcriptional coactivator for steroid receptors and various transcription factors (7, 8), CARM1 has since been recognized for its critical contributions to multiple cellular processes, including gene expression regulation, mRNA splicing fidelity, and embryonic development (9, 10, 11, 12, 13, 14).

CARM1's primary substrate *in vivo* is histone H3, with specific methylation occurring at arginine residues R17 and R26 (15, 16). The resultant modifications, particularly H3R17 methylation (H3R17me2a), are instrumental in transcriptional activation through various mechanisms, including the recruitment of coactivators and chromatin remodeling factors. Chromatin immunoprecipitation (ChIP) studies have revealed CARM1's targeted recruitment to specific gene promoters, where it deposits H3R17 dimethylation marks to modulate gene expression (15, 16). The essential nature of CARM1's methyltransferase activity has been demonstrated through studies utilizing enzyme-dead knock-in models and CARM1 mutants, highlighting its indispensable role in gene promoter activation (10, 11). The pathological implications of CARM1 dysregulation extend across multiple diseases. In cancer biology, CARM1 has been implicated in various malignancies (17, 18, 19, 20, 21, 22, 23, 24), with particular emphasis on its role in estrogen-induced proliferation in breast cancer cells (12). A notable mechanism involves the asymmetric arginine methylation of BRG1-associated factor 155 (BAF155), a SWI/SNF core subunit and CARM1 substrate, which shows elevated levels in human breast cancer. The methylated BAF155 is recruited to c-Myc pathway genes, regulating metastatic pathways in triple-negative breast cancer cells, thus exemplifying how CARM1-mediated methylation of nonhistone substrates can promote tumor progression and metastasis (25). In the realm of metabolic diseases, CARM1's significance has become increasingly apparent. Elevated CARM1 expression has been observed in type II diabetes, diabetic retinopathy, and obesity (14, 26, 27), with recent studies illuminating its crucial role in liver pathophysiology, lipid metabolism, and adipogenesis (28, 29, 30). In hepatocytes, CARM1-mediated H3R17me2a enhances the transcription of key gluconeogenic enzymes, phosphoenolpyruvate carboxy kinase (PCK1) and glucose-6-phosphatase (G6PC), thereby promoting hepatic gluconeogenesis and contributing to hyperglycemia (31). Furthermore, CARM1's involvement in diabetic retinopathy occurs through its recruitment to p53-responsive elements of apoptotic target gene promoters, where it catalyzes H3R17 dimethylation, leading to retinal pigment epithelium apoptosis under hyperglycemic conditions (14). The therapeutic potential of targeting CARM1 has been demonstrated through pharmacological interventions. Inhibitors such as 2,3,7,8-tetrahydroxy-benzopyranol[5,4,3-cde]benzopyran-5,10-dione (TBBD) and ellagic acid have shown promise in attenuating retinal pigment epithelium cell apoptosis during diabetic retinopathy progression by blocking CARM1 activity and H3R17 dimethylation (32). While several CARM1 inhibitors have been identified (33, 34, 35, 36, 37), their specific effects on histone methylation marks warrant further investigation.

In parallel, metformin, a widely prescribed first-line medication for type II diabetes, has demonstrated remarkable efficacy in reducing mortality and disease complications (38, 39, 40, 41, 42). Beyond its established role in diabetes management, metformin has shown promise in inhibiting tumor growth, metastasis, and cancer cell migration across multiple cancer types (43, 44, 45, 46, 47, 48). Epidemiological studies suggest its protective effects against various malignancies in diabetic patients (49, 50, 51). Despite its widespread use and documented benefits, the precise therapeutic targets and epigenetic mechanisms underlying metformin's effects remain poorly understood, particularly regarding its potential influence on histone modifications implicated in metabolic diseases.

Given the convergence of CARM1's role in both cancer and metabolic disorders, and metformin's diverse therapeutic effects, we sought to investigate whether CARM1 serves as a potential therapeutic target for metformin. Our study aims to elucidate how metformin influences chromatin dynamics through CARM1, both *in vitro* and *in vivo*, with particular focus on its effects on key gluconeogenic enzymes. This investigation may provide crucial insights into the epigenetic mechanisms underlying metformin's therapeutic actions and potentially reveal new therapeutic strategies for both metabolic disorders and cancer.

## Results

### Asymmetric arginine posttranslation modification and metformin mimicry

**Figure 1:**
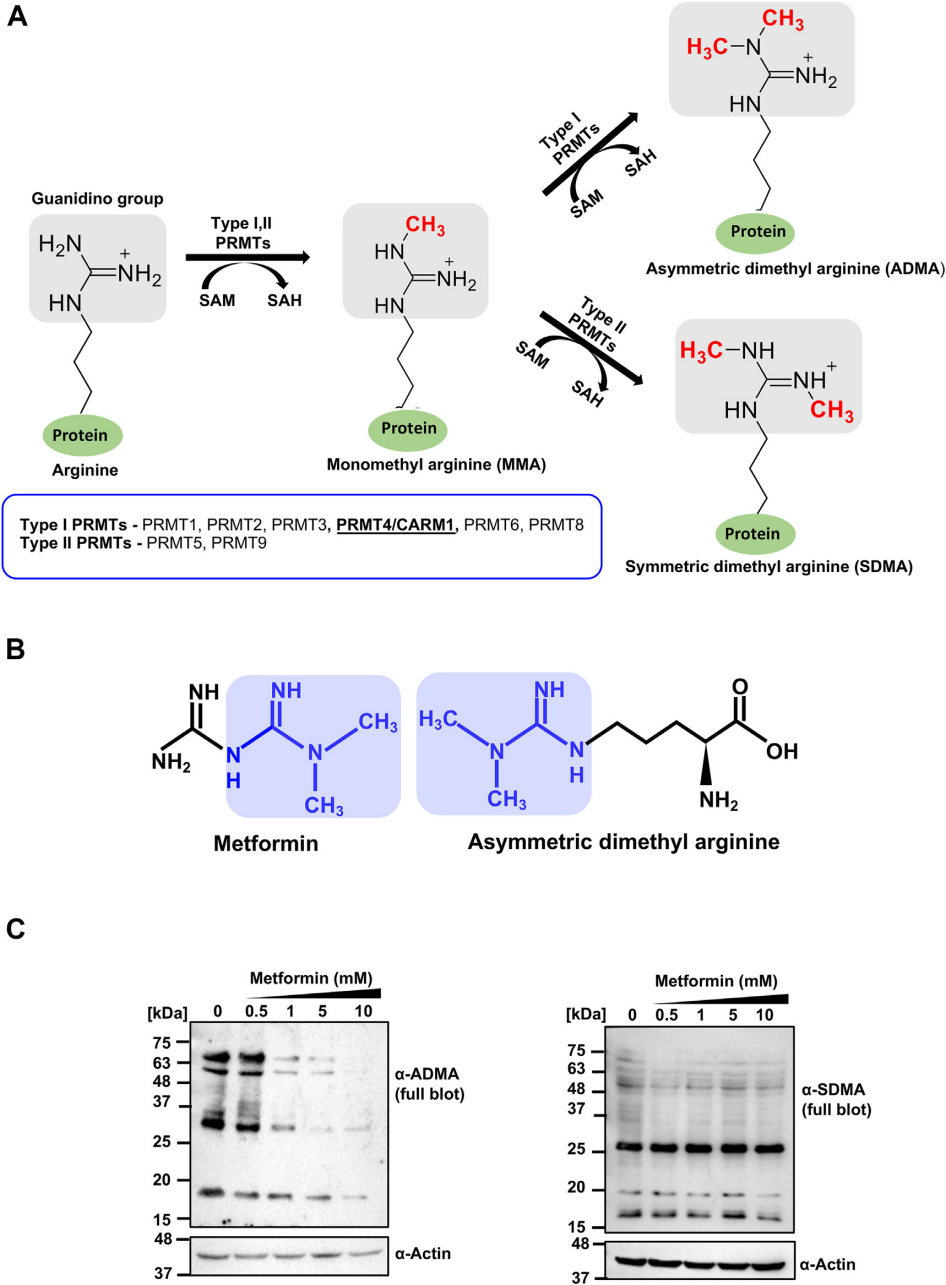
**Metformin is an inhibitor of type I methyltransferase enzymes**. *A*, the enzymatic process of methyltransferase, as facilitated by protein arginine methyltransferases (PRMTs) of types I and II, involves the transfer of a methyl group from the cofactor S-adenosyl-L-methionine to the guanidino nitrogen atom of arginine. Type I PRMTs (including PRMT1, 2, 3, 4, 6, and 8) are responsible for catalyzing asymmetric dimethylarginine, and type II PRMTs (PRMT5 and 9) are involved in the catalysis of symmetric dimethylarginine. *B*, metformin exhibits structural similarity to the asymmetric dimethylarginine, a product catalyzed by type I PRMTs. *C*, Western blot showing level of asymmetric and symmetric dimethylation, in HepG2 cells following a 12-h treatment with varying concentrations of metformin (0.5, 1, 5, and 10 mM).

Posttranslational modifications of arginine include the addition of methyl groups, resulting in monomethyl arginine or dimethylarginine. Asymmetric dimethylarginine (ADMA) arises from the addition of two methyl groups to the same guanidine nitrogen atom, catalyzed by type I PRMTs. In contrast, symmetric dimethylarginine results from methylation on each terminal nitrogen atom by type II PRMTs (Fig. 1*A*).

Metformin, a widely used antidiabetic drug, structurally resembles asymmetrically dimethylated arginine. Comparative analysis shows that the dimethylated guanidino group of metformin mimics asymmetrically dimethylated arginine (Fig. 1*B*). This structural similarity suggests that metformin might mimic the product of ADMA and potentially bind to the active site of type I PRMTs, inhibiting their product formation. To test this hypothesis, HepG2 cells were treated with varying doses of metformin. Western blot analysis revealed a significant reduction in overall asymmetric dimethylation levels in cell lysates, while symmetric dimethylation remained unaffected (Fig. 1*C*). These results indicate that metformin selectively targets the substrate-binding site of type I PRMTs, leading to decreased formation of asymmetric dimethylation products.

### Metformin suppresses human CARM1 methyltransferase activity *in vitro* in a dose-dependent manner

**Figure 2:**
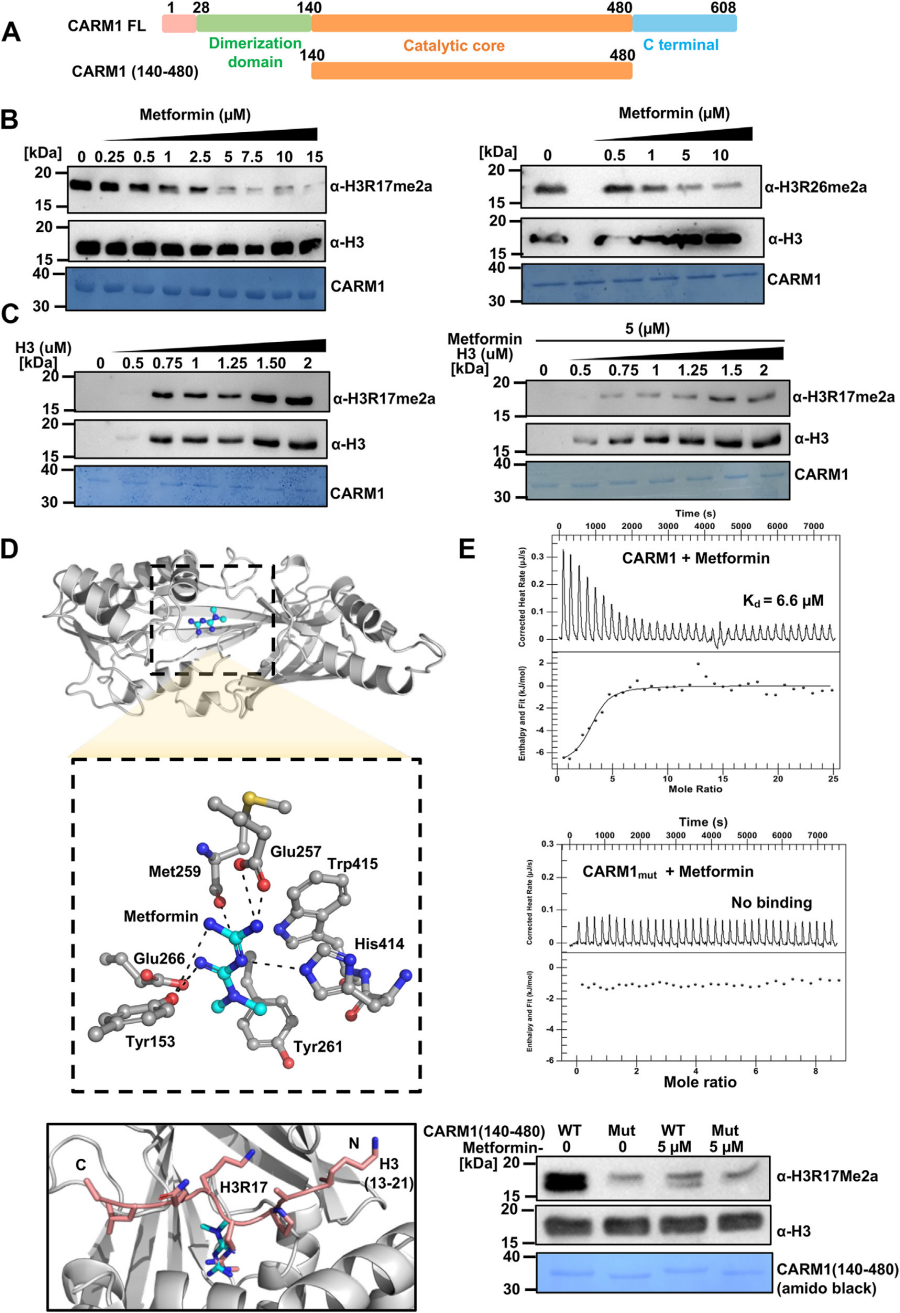
**Inhibition of CARM1 methyltransferase activity by metformin *in vitro*.***A*, domain architecture of human full-length CARM1 and its catalytic core (140–480). *B*, *in vitro* methyltransferase assay with purified CARM1 (140–480) and recombinant histone H3 with increasing concentration of metformin. Samples were analyzed by Western blotting and probed with anti H3R17me2a and H3R26me2a antibodies. *C*, *in vitro* methyltransferase assay with increasing substrate (recombinant H3) concentration in the absence and presence of metformin (5 μM). The level of H3R17me2a was monitored by Western blotting analysis. *D*, docking studies to show the position of metformin (in ball and stick model in *cyan* and *blue*) in the substrate-binding pocket of CARM1(PDB ID 2V7E) and superimposition of H3 peptide (13–21, *pink*) bound CARM1 with metformin occupy the same binding pocket. *E*, isothermal titration calorimetry showing metformin binding to CARM1 WT and quadruple CARM1mut and HMTase assay using recombinant histone H3 with CARM1 WT and quadruple CARM1mut. CARM1, coactivator-associated arginine methyltransferase 1; HMT, histone methyltransferase; PDB, Protein Data Bank.

CARM1, a major type I PRMT, generates asymmetrically dimethylated arginine on both histone and nonhistone substrates in eukaryotic cells. The full-length human CARM1 protein, comprising 608 amino acids, contains a central methyltransferase domain crucial for its catalytic function. We investigated whether metformin can inhibit CARM1 enzymatic activity. To assess the impact of metformin on CARM1 enzymatic activity *in vitro*, we isolated and purified the catalytic domain CARM1 (140–480), in an N-terminal GST-tagged vector for subsequent *in vitro* analyses (Fig. 2*A*). Utilizing this catalytic domain construct and recombinant human histone H3 as a specific substrate for CARM1, we conducted *in vitro* methyltransferase assays. We observed a dose-dependent decrease in H3R17me2a and H3R26me2a marks with increasing concentrations of metformin (Fig. 2*B*), indicating that metformin inhibits CARM1-mediated histone H3 methylation. To explore whether metformin's inhibitory effect is contingent upon substrate concentration, we performed experiments with a constant metformin concentration and varying histone H3 substrate concentrations. We observed diminished formation of H3R17me2a even at higher concentrations of histone H3 (Fig. 2*C*). These observations suggest a competitive mode of inhibition by metformin, emphasizing its ability to impede CARM1-mediated histone methylation. We utilized a machine learning-based docking tool to interpret the exact binding site of metformin to CARM1 (Protein Data Bank [PDB] ID 4IKP). Docking of metformin using DiffDock yielded several binding poses, with the top-ranked pose selected for detailed analysis (52). The binding affinity of the best pose was found to be −3.8 kcal/mol, indicating a strong interaction between metformin and the active site of CARM1. Key interactions included hydrogen bonds between the guanidino groups of metformin and the side chains of residues Glu-266, Glu-257, Tyr-153, and His-414. The dimethyl group of metformin is stabilized through a hydrophobic pocket formed by Tyr-261 and Trp-415 (Fig. 2*D*, top). Superposition of the metformin dock structure with the H3 (13–21) bound CARM1 cocrystal structure shows that metformin occupies the same pocket as the arginine 17 residue of histone H3 (Fig. 2*D*, bottom). To evaluate the potential physical interaction between metformin and CARM1 (140–480), we performed isothermal titration calorimetry (ITC). Thermogram analysis revealed that CARM1 demonstrated a low micromolar affinity for metformin (K_d_ = 6.6 μM) (Fig. 2*E*, top). Next, we were intrigued to look into the binding site of metformin by mutational studies. We mutated the four residues (Glu-266, Glu-257, Tyr-153, and His-414) of CARM1 to alanine and denoted this quadruple mutant as CARM1mut (Y153A/E257A/E266A/H414A). These residues were predicted to be important for metformin interaction, as revealed by the docking analysis (Fig. 2*D*). Isothermal calorimetry was done with the quadruple mutant and metformin and no heat change was observed (Fig. 2*E*, middle). Next, histone methyltransferase assay was done to ascertain the activity of the enzyme, due to the mutation. Indeed, the mutation in residues (Tyr-153, Glu-257, Glu-266, and His-414) led to a decrease in H3R17me2a levels substantially in comparison to WT CARM1 (Fig. 2*E*, bottom). Overall, this suggests that the metformin binding site is same as the substrate-binding site of CARM1. Cocrystal data from previous studies also show that the aforementioned residues in CARM1 are crucial for substrate binding, which further support our finding (53, 54).

### Metformin inhibits CARM1 methyltransferase activity in hepatic cells

**Figure 3:**
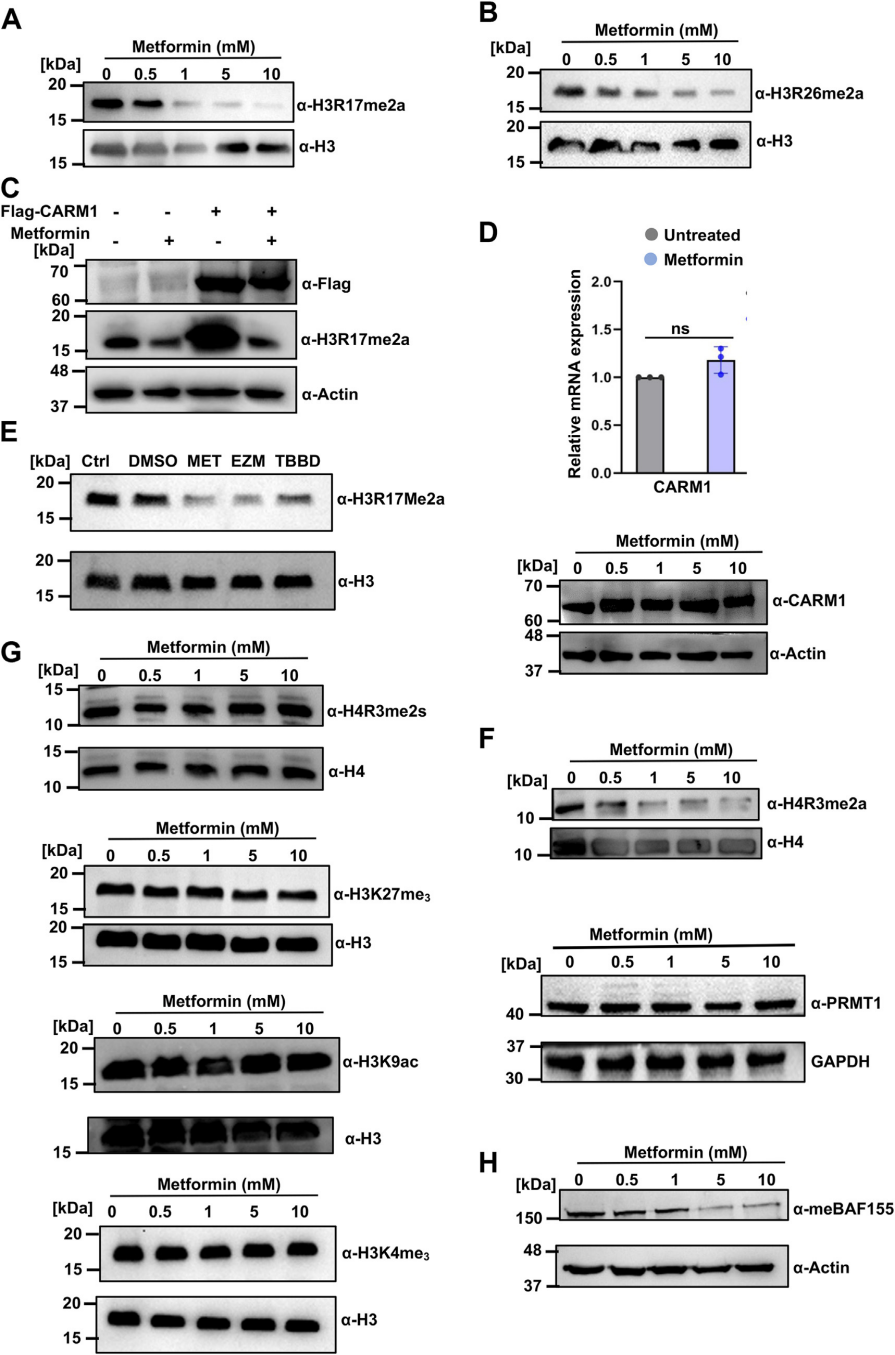
**Metformin inhibits methyl transferase activity of CARM1 in hepatic cells**. Western blot analysis from HepG2 cells treated with increasing concentrations of metformin for 12 h and probed with (*A*) anti H3R17me2a antibody and (*B*) anti H3R26me2a antibody. *C*, FLAG CARM1 was overexpressed in HepG2 cells and subsequently treated with 10 mM metformin for 12 h and probed with anti H3R17me2a antibody. *D*, expression of CARM1 upon metformin treatment in HepG2 cells monitored in RNA level and protein level. *E*, HepG2 cells treated with appropriate concentration of different CARM1 inhibitors *i.e.*, EZM2302 (100 nM) and TBBD (10 μM) for 24 h and metformin (5 mM) for 12 h and probed with anti H3R17me2a antibody. *F*, Western blot showing the level of histone H4R3me2a, catalyzed by PRMT1, a type I PRMT and expression of PRMT1 enzyme in metformin treated HepG2 cells. *G*, Western blot showing levels of histone H4R3me2s, H3K27me3, H3K4me3, and H3K9ac upon metformin treatment. *H*, methylation status of BAFF155 upon metformin treatment as observed by Western blotting. BAF155, TBBD, 2,3,7,8-tetrahydroxy-benzopyranol[5,4,3-cde]benzopyran-5,10-dione; BRG1-associated factor 155; CARM1, coactivator-associated arginine methyltransferase 1; EZM2302, 2-[2-[2-chloro-5-[(2R)-2-hydroxy-3-(methylamino) propoxy] phenyl]; PRMT, protein arginine methyltransferase.

We investigated whether metformin could inhibit CARM1 methyltransferase activity in cellular context. In human hepatic cell line, HepG2 cells treated with metformin exhibited a dose-dependent reduction in the asymmetric dimethylation of residues R17 and R26 on histone H3 (H3R17me2a and H3R26me2a) (Fig. 3, *A* and *B*). This result aligns with our previous *in vitro* findings. To further explore, we transiently overexpressed FLAG-tagged CARM1 in HepG2 cells, which resulted in elevated H3R17me2a levels. Metformin treatment subsequently led to a significant reduction in H3R17me2a levels (Fig. 3*C*), indicating that metformin impairs histone methylation *via* CARM1 inhibition. To ascertain whether the observed reduction in methylation marks was due to changes in CARM1 expression, we performed quantitative real-time PCR and protein level assessments on metformin-treated cell lysates. Results showed no significant changes in CARM1 expression at the RNA or protein levels (Fig. 3*D*), confirming that metformin does not affect CARM1 expression itself. The effectiveness of metformin inhibition of CARM1 was compared with other well-known inhibitors of CARM1 *i.e.*, TBBD and 2-[2-[2-chloro-5-[(2R)-2-hydroxy-3-(methylamino) propoxy] phenyl] (EZM2302), which are known specific inhibitors of CARM1 (32, 33). Both the compounds reduced H3R17me2a levels in nanomolar and micromolar concentrations, respectively. These inhibitors served as a positive control for metformin inhibition (Fig. 3*E*). To gain insight about the specificity of metformin on other members of the type I PRMT family and its effect on asymmetric dimethylation of histone residues, we tested its effect on PRMT1-mediated H4R3me2a mark. Surprisingly, metformin treatment reduced H4R3me2a levels (Fig. 3*F*, top), suggesting that metformin has an affinity for type I PRMTs (55). Expression of PRMT1 was also unchanged upon metformin inhibition, as seen in Western blot (Fig. 3*F*, bottom). We then questioned whether metformin affects type II PRMTs, specifically looking at PRMT5-mediated symmetric dimethylation of H4R3 (H4R3me2s). Western blot analysis revealed unchanged levels of H4R3me2s (Fig. 3*G*, top), indicating that metformin exhibits specificity for type I PRMTs and does not inhibit type II PRMTs. Furthermore, we assessed the effect of metformin on histone lysine methyltransferases and histone acetyltransferases. We found no change in lysine methylation (H3K27me3 and H3K4me3) or acetylation (H3K9ac) marks (Fig. 3*G*, bottom), indicating that metformin does not affect marks catalyzed by these enzymes. Besides histone H3, CARM1 methylates various nonhistone substrates, such as BAF155, a subunit of the SWI/SNF core complex implicated in several cancers, methylated by CARM1 at arginine 1064 (9, 54). Metformin treatment resulted in reduced asymmetrical dimethylation of BAF155 at R1064 (Fig. 3*H*), demonstrating that metformin effectively blocks CARM1's methyltransferase activity on both histone and nonhistone substrates.

### Metformin inhibits CARM1 in mouse primary hepatocytes and *in vivo*

**Figure 4:**
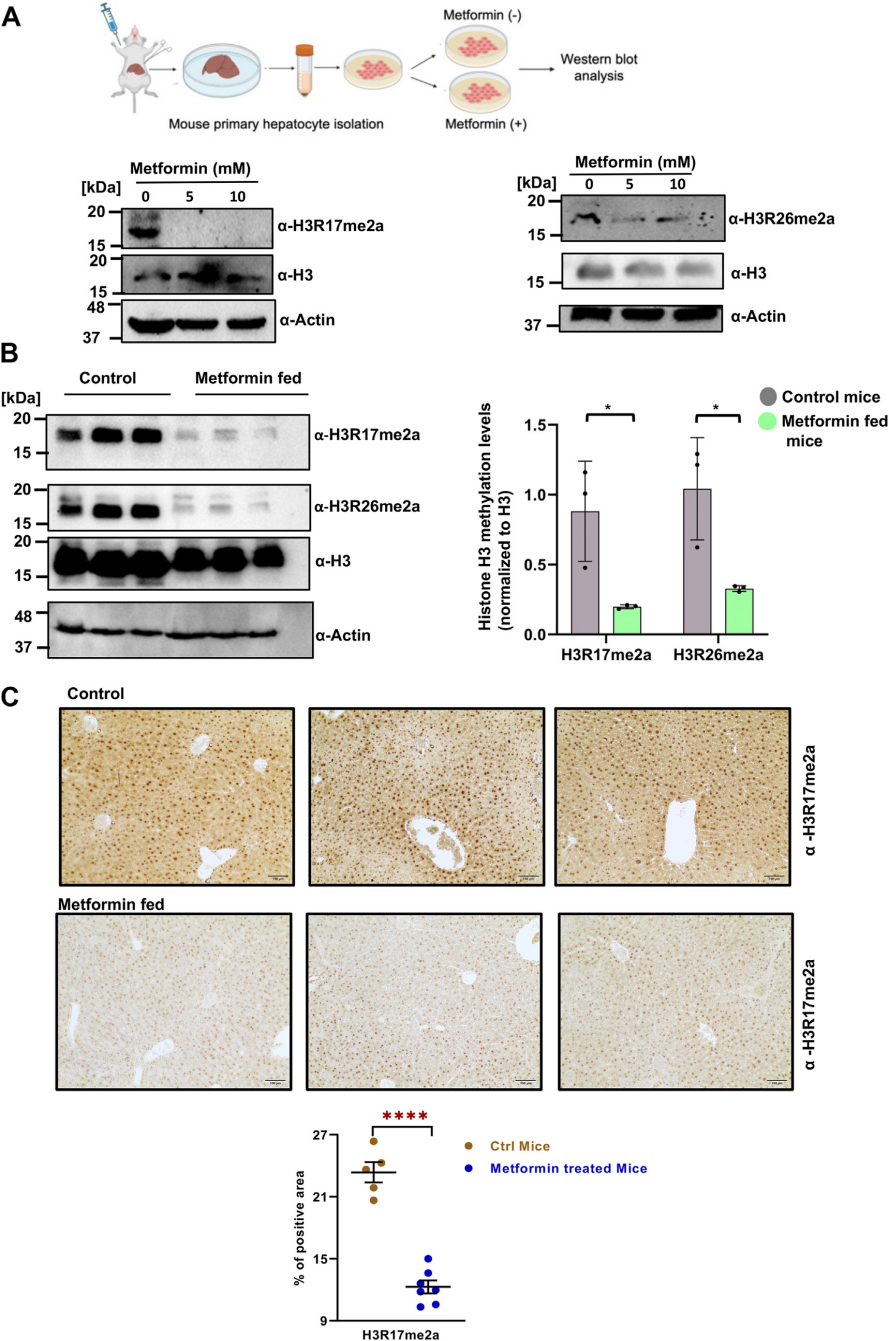
***In vivo* study of metformin mediated CARM1 inhibition**. *A*, mouse primary hepatocyte was treated with metformin (5 mM and 10 mM) for 12 h and analyzed by Western blotting using anti-H3R17me2a and anti-H3R26me2a antibodies. *B*, Western blot images and quantification of band intensities of histone H3R17me2a mark in the liver tissue of three representatives from the control group and metformin fed mice. Data points are shown in the plot and significant differences (*p* < 0.05) are marked with *asterisks*. *C*, images of immunohistochemical staining of liver sections with anti H3R17me2a antibody (the scale bars represent 100 μm) of control (*top*) and metformin fed (*bottom*) group of mice and quantitative analysis of H3R17me2a levels derived from immunohistochemical images. CARM1, coactivator-associated arginine methyltransferase 1.

To determine the inhibitory effect of metformin on mouse model, we isolated primary hepatocytes from C57BL/6 mice and treated them with metformin (Fig. 4*A*, top). CARM1 catalyzed H3R17me2a and H3R26me2a levels were found significantly decreased in metformin-treated hepatocytes as compared to untreated cells, as documented by Western blot analysis (Fig. 4*A*, bottom). Changes in H3R17me2a and H3R26me2a levels were then assessed in liver specimens obtained from metformin-fed and control C57BL/6 mice. Metformin-fed mice liver showed significant decrease in histone H3R17me2a and H3R26me2a modification levels as compared to control, shown in the Western blot image (Fig. 4*B*, left). Quantification of band intensities was determined by ImageJ software (https://imagej.net/ij/index.html) which represents the decrease in H3R17me2a in metformin-treated samples (Fig. 4*B*, right). Paraffin-embedded liver sections of metformin fed and control mice were subjected to immunohistochemical analysis, and representative images show significant decrease of nuclear H3R17me2a levels in metformin-fed mice liver samples. Images of the liver sections of metformin fed and control mice were quantified (Fig. 4*C*). This suggests that metformin was able to deplete H3R17me2a levels, mediated by CARM1, in mouse model.

### Metformin downregulates expression of CARM1-dependent gluconeogenic genes *in vitro* and *in vivo*

**Figure 5:**
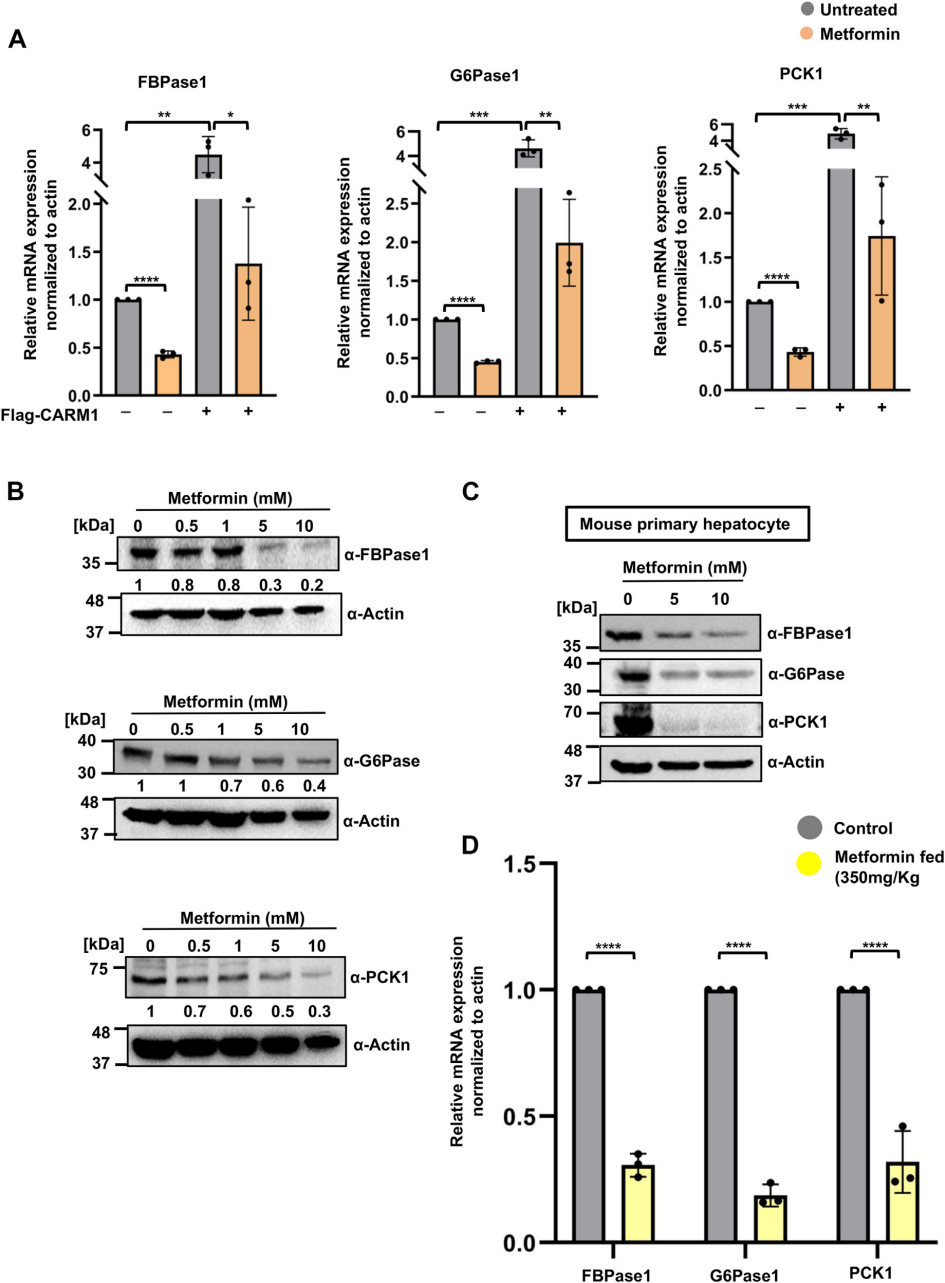
**Metformin treatment of hepatic cells leads to the downregulation of gluconeogenic gene expression.***A*, cells were treated with metformin (10 mM) for 12 h in control and CARM1 overexpressed condition and relative mRNA expression of gluconeogenic genes, FBPase1, G6Pase, and PCK1 was quantified by qRT-PCR. In panels (*A*), error bars indicate standard deviation, n = 3. *p*-values were calculated using unpaired Student's *t* test. ∗*p* < 0.05; ∗∗*p* < 0.01; ∗∗∗*p* < 0.001; and ∗∗∗∗*p* < 0.0001. *B*, protein expression of gluconeogenic enzymes FBPase1, G6Pase, and PCK1 was checked upon metformin treatment by Western blot analysis. Band intensities were quantified by ImageJ software and represented numerically, placed below the blots. *C*, protein levels of G6Pase, FBPase1, and PCK1 were analyzed by Western blot in mouse primary hepatocyte upon metformin treatment. *D*, relative mRNA expression of gluconeogenic genes, FBPase1, G6Pase, and PCK1 in the liver samples of metformin fed and control mice was quantified by qRT-PCR. Error bars indicate standard deviation, n = 3. *p*-values were calculated using unpaired Student's *t* test. ∗*p* < 0.05; ∗∗*p* < 0.01; ∗∗∗*p* < 0.001; and ∗∗∗∗*p* < 0.0001. CARM1, coactivator-associated arginine methyltransferase 1; FBPase 1, fructose 1,6-bisphosphatase 1; PCK1, phosphoenolpyruvate carboxy kinase 1; qRT-PCR, quantitative RT-PCR.

We investigated potential changes in the expression of downstream target genes of CARM1 as a consequence of its inhibition. We assessed whether metformin impacts the expression levels of key gluconeogenic enzymes, G6Pase1, fructose 1,6-bisphosphatase 1 (FBPase1), and PCK1, which are established downstream targets of CARM1 (31). Through quantitative RT-PCR (qRT-PCR), we observed up to 50% reduction in the expression of these genes upon treatment with metformin. Additionally, upon transient transfection with FLAG-CARM1, the expression of these genes was elevated; however, this elevation was significantly reversed after metformin treatment (Fig. 5*A*). To further confirm the inhibitory effect on gene expression at the protein level, we performed Western blot analysis of cell lysates from metformin-treated cells. It was observed that the expression of gluconeogenic enzymes (*i.e.*, G6Pase1, FBPase1, and PCK1) at the protein level were significantly minimized, in the presence of metformin, which also corroborates the qRT-PCR data (Fig. 5*B*). We extended our analysis to mouse primary hepatocytes treated with metformin (5 mM and 10 mM) for 12 h. Western blot results showed a substantial reduction in the protein levels of these gluconeogenic enzymes, further supporting our earlier observations (Fig. 5*C*). We then assessed whether metformin inhibits CARM1 *in vivo*, in mouse model. qRT-PCR analysis of metformin fed and control mouse liver samples showed significant downregulation of gluconeogenic gene expression in treated mice relative to control mice (Fig. 5*D*). This corroborates to the antihyperglycemic effect of metformin at the cellular level. Overall, these experiments suggest that metformin leads to decreased gluconeogenic gene transcription *via* inhibition of CARM1, at both the mRNA and protein level. This highlights metformin's potential as a therapeutic agent in modulating gluconeogenesis.

### Effect of metformin on CARM1-mediated H3R17me2a marks in gluconeogenic gene promoters

**Figure 6:**
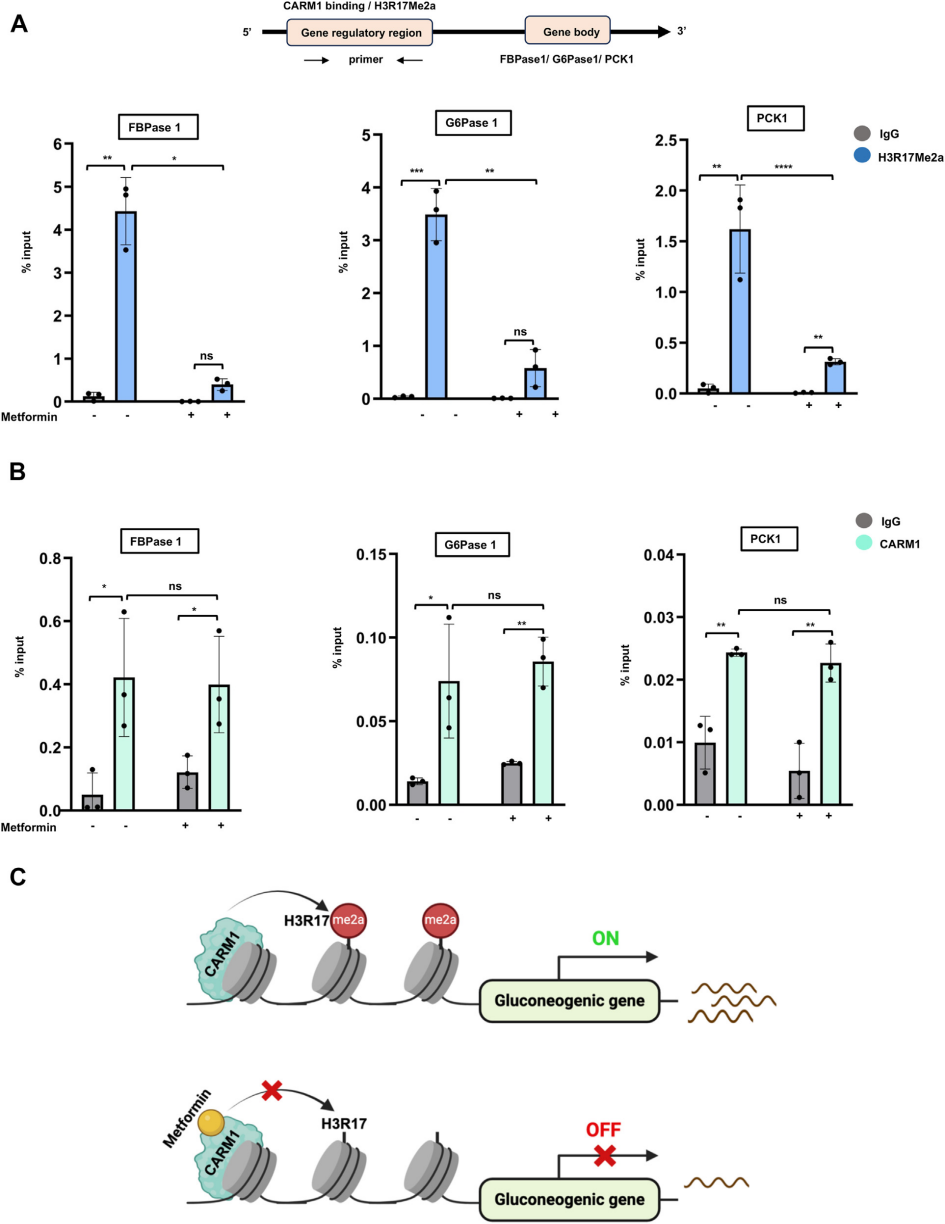
**Metformin reduces histone H3R17me2a levels from gluconeogenic gene promoters**. Chromatin immunoprecipitation from untreated and metformin (10 Mm) treated hepatic cells was performed using (*A*) anti H3R17me2a and (*B*) anti CARM1 antibodies, in CARM1 overexpressed and control condition, in G6Pase, FBPase1 and PCK1 promoters. All data sets were taken from three biological replicates. β actin was used as a negative control. Error bars indicate standard deviation, n = 3. *p*-values were calculated using one-way ANOVA. ∗*p* < 0.05; ∗∗*p* < 0.01; ∗∗∗*p* < 0.001; ∗∗∗∗*p* < 0.0001; and ns, nonsignificant *p* > 0.05. *C*, schematic diagram of CARM1 inhibition by metformin that leads to suppression of CARM1 induced overexpression of gluconeogenic genes. CARM1, coactivator-associated arginine methyltransferase 1; FBPase 1, fructose 1,6-bisphosphatase 1; PCK1, phosphoenolpyruvate carboxy kinase 1.

To test whether CARM1 inhibition preferentially affects H3R17me2a mark on gluconeogenic gene promoters, leading to aberrant transcriptional silencing, we treated HepG2 cells with 10 mM of metformin and carried out ChIP experiments. As predicted, we observed a decrease in H3R17me2a levels in metformin treated cells as compared to the untreated cells (Fig. 6*A*). Next, to rule out the fact that, whether metformin affected CARM1 recruitment to these gene regulatory regions, we conducted ChIP experiments with CARM1 specific antibody, to confirm the status of CARM1 recruitment on the gluconeogenic gene promoters. ChIP analysis in metformin-treated cells indicated that CARM1 recruitment to gluconeogenic gene promoters remained unchanged (Fig. 6*B*). Mechanistically, CARM1 binds to regulatory regions upstream of genes like G6Pase, FBPase, and PCK1, maintaining H3R17 asymmetric dimethylation, a mark associated with gene activation, under normal conditions. In contrast, metformin-mediated inhibition of CARM1 reduces these histone arginine methylation marks, downregulating gluconeogenic gene expression. This suggests that metformin modulates the H3R17 dimethylation by CARM1 in gene regulatory regions which is crucial for altering transcription rates and might control the associated phenotypic changes (Fig. 6*C*).

### Impact of CARM1 overexpression on gluconeogenic activity and its rescue by metformin

**Figure 7:**
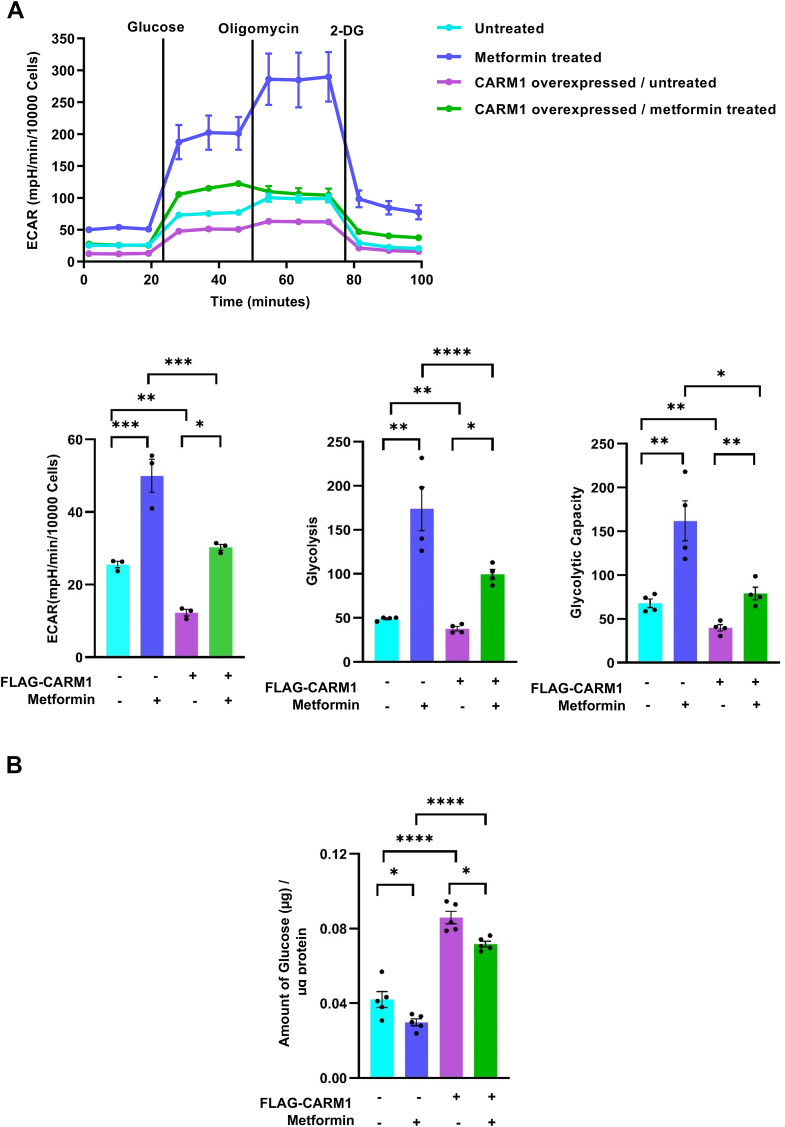
**Metformin treatment alters CARM1 mediated deregulation of glucose metabolism.***A*, representation of extracellular acidification rate (ECAR) measurement in CARM1 overexpressed condition, in the presence and absence of 10 mM metformin in HepG2 cells. Quantification of extracellular acidification rate, glycolysis, and glycolytic capacity in CARM1 overexpressed condition, in the presence and absence of 10 mM metformin in HepG2 cells. An unpaired student *t* test was performed to analyze the *p*-value significance (∗*p* < 0.05; ∗∗*p* < 0.01; ∗∗∗*p* < 0.001; ∗∗∗∗*p* < 0.0001; and ns, nonsignificant (*p* > 0.05)) for the statistical analyses. The error bar represents the standard error of the mean (SEM). *B*, glucose production assay in CARM1 overexpressed condition, in the presence and absence of 10 mM metformin in HepG2 cells. One-way ANOVA was performed to analyze the *p*-value significance (∗*p* < 0.05; ∗∗*p* < 0.01; ∗∗∗*p* < 0.001; ∗∗∗∗*p* < 0.0001; and ns, nonsignificant (*p* > 0.05)) for the statistical analyses. The error bar represents the standard error of the mean (SEM). CARM1, coactivator-associated arginine methyltransferase 1.

Next, we examined the changes in the metabolic state in HepG2 cell induced by metformin. Given the reciprocal regulation between gluconeogenesis and glycolysis within the cell (56) and experiments, which suggest that CARM1 leads to increased gluconeogenesis, we postulated that overexpression of CARM1 might lead to glycolytic suppression, which could be rescued by metformin. Using a real-time metabolic assay measuring extracellular acidification rate (ECAR), a direct indicator of glycolytic activity, we found that CARM1 overexpression markedly reduced glycolysis compared to controls, as indicated by decreased ECAR levels (Fig. 7*A*). Subsequent treatment with metformin significantly increased ECAR (*i.e.*, glycolysis and glycolytic capacity) in both untransfected and CARM1-overexpressing cells, which is an indicator of increased uptake of free glucose present in the cell (Fig. 7*A*). These findings underscore that metformin effectively reverses the glycolytic suppression induced by CARM1 overexpression, highlighting its potential to mitigate the hyperglycemic effects associated with elevated CARM1 activity. These results prompted us to measure the rate of glucose production, which is a robust indicator of gluconeogenic activity in the cell. Consistent with our hypothesis, CARM1 overexpression resulted in significantly higher glucose concentrations compared to untransfected cells, indicative of increased gluconeogenic activity (Fig. 7*B*). This effect was substantially reduced upon metformin treatment. These results provide compelling evidence that CARM1 overexpression suppresses glycolysis and, in-turn, elevates gluconeogenesis, contributing to increased glucose levels, which can be effectively countered by metformin through mechanisms involving inhibition of CARM1 methyltransferase activity, specifically histone H3 methylation.

## Discussion

**Figure 8:**
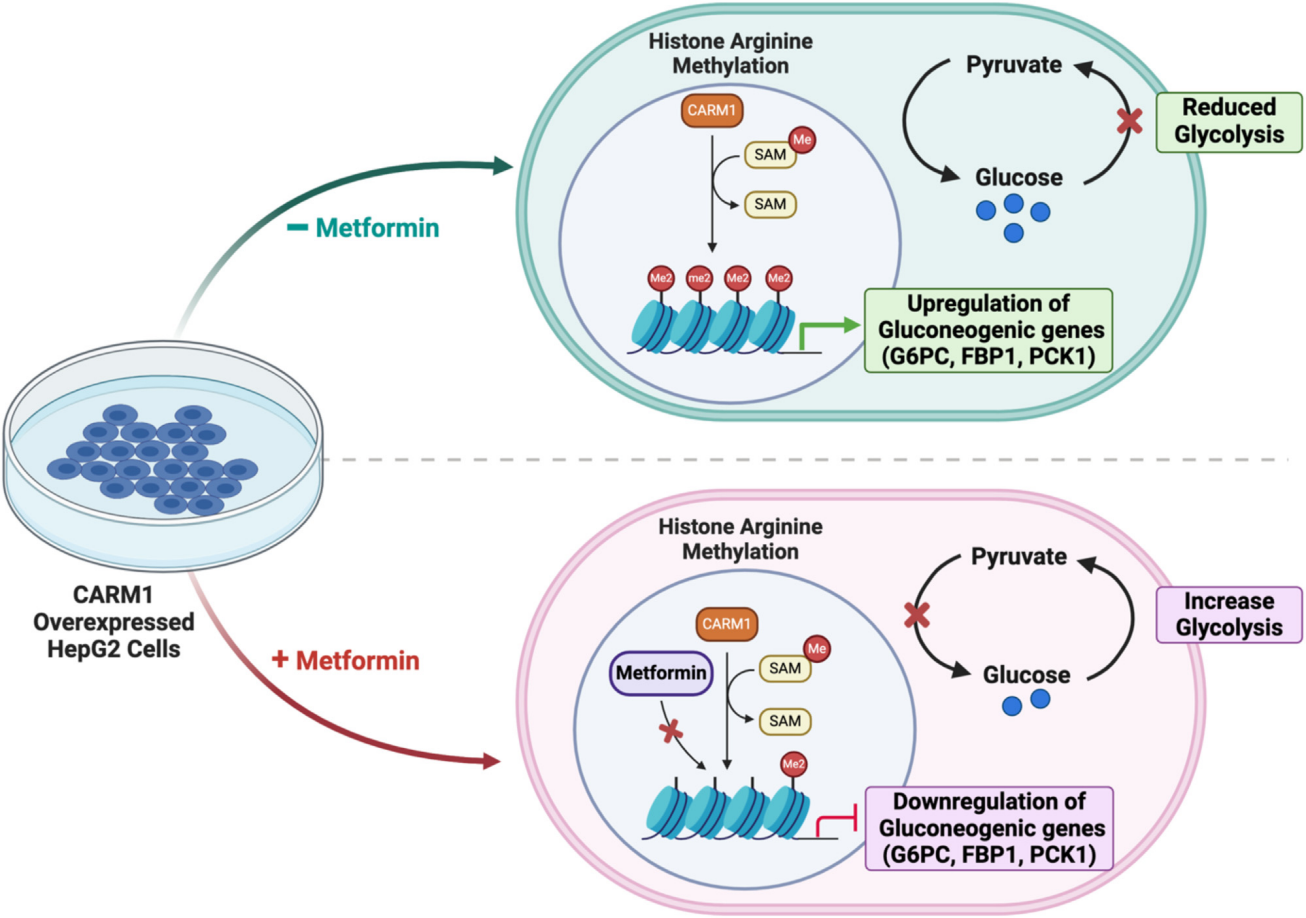
**Schematic representation of metformin mediated suppression of CARM1 methylation activity at the gluconeogenic gene promoter.** CARM1, coactivator-associated arginine methyltransferase 1.

Our study reveals the intricate interplay between arginine posttranslation modification and the antidiabetic drug metformin, emphasizing its potential role in inhibiting the methyltransferase activity of CARM1. Given metformin's structural resemblance to asymmetrically dimethylated arginine, we propose that metformin acts as a competitive inhibitor of type I PRMT, specifically CARM1. Biochemical experiments confirmed that metformin treatment in human hepatic cell lines led to a decrease in overall asymmetric dimethylated arginine levels. A methyltransferase assay demonstrated metformin-mediated, dose-dependent suppression of CARM1's catalytic activity *in vitro*, resulting in reduced CARM1-specific histone H3 methylation marks (H3R17me2a and H3R26me2a). Molecular docking studies showed that metformin's binding closely mirrors the arginine position of histone H3R17 in the CARM1 active site, reinforcing its role as a competitive inhibitor. Further biophysical studies revealed that metformin binds to CARM1 (140–480) with low micromolar affinity but, failed to interact when residues crucial for its binding were mutated suggesting that disruption of these hydrogen bond interactions abolished the binding of metformin to CARM1. In the absence of metformin, the CARM1mut construct was unable to catalyze the dimethylation of histone H3 at arginine 17 (H3R17me2a), a known CARM1 substrate. This indicates that the mutations in the identified binding site residues rendered CARM1 catalytically inactive. These results provide further support for the conclusion from our molecular docking studies that metformin competitively inhibits CARM1 by occupying its active site. Our study demonstrates that metformin is a potent inhibitor of CARM1 in human hepatic cells and *in vivo*. Treated HepG2 cells and mouse liver exhibit reduced H3R17me2a and H3R26me2a levels as compared to control. Similar phenomenon was observed in paraffin-embedded immunostained mouse liver sections. Transient overexpression of FLAG-tagged CARM1 vastly enhanced H3R17me2a levels, a change that was effectively reversed after subsequent treatment with metformin. This result suggests that the observed impairment of histone methylation marks can be attributed specifically to CARM1 inhibition. We also showed that metformin effectively reduces asymmetrically dimethylated BAF155 levels, indicating that metformin's inhibitory effects extend to nonhistone substrates as well. Furthermore, our study revealed that metformin altered the asymmetric dimethylation of arginine position 3 of histone H4, mediated by PRMT1 (another type I PRMT), whereas the symmetric dimethylation of the same residue, catalyzed by type II PRMT (PRMT5), remained unchanged. The similarity between type I PRMT-mediated ADMA and the methylated guanidino group of metformin may explain the specificity of metformin over type I PRMTs. This selective inhibition highlights metformin's preference for type I PRMTs over type II PRMTs. A notable finding is that metformin does not alter CARM1 or PRMT1 expression in RNA and protein levels, ruling out other possible causes behind the observed inhibitory effects. Type I PRMTs are implicated in various diseases (56, 57, 58), and understanding how metformin regulates them may provide deeper insights into its mode of action. A key finding is that metformin downregulates the expression of gluconeogenic genes, such as G6Pase1, PCK1, and F1,6BPase1, in hepatic cell line and *in vivo*, potentially reducing the protein levels of these enzymes. By chromatin immunoprecipitation studies, we observed reduced CARM1 catalyzed histone H3R17me2a marks on the promoters of these specific genes, which are known activation marks (16, 59), which confirms the downregulation of these genes *via* CARM1 inhibition. Further, we noted no change in CARM1 recruitment to these specific gene promoters, due to metformin treatment, in HepG2 cell line. To determine the downstream effects of reduced gluconeogenic gene expression, we analyzed glycolytic rates and glucose production levels in live cells. As expected, CARM1 overexpression led to a decreased glycolytic rate and increased glucose levels, which were effectively reversed by metformin. These results underscore metformin's potential therapeutic benefits as an antihyperglycemic drug. A proposed model on the mode of action of metformin in hepatic cells has been illustrated (Fig. 8). Our study explores the ability of metformin to alter the epigenetic landscape, thus acting as an epigenetic drug. We also identify CARM1, a histone modifier, as a target for metformin which paves the way to further investigate its role in CARM1 mediated pathways.

## Experimental procedures

### Cloning, expression, and purification

The human CARM1 methyltransferase domain (residues 140–480) was cloned into the pGEX-6p-1 vector (GE HealthCare; catalog no.: GE28-9546-48) for bacterial expression, incorporating an N-terminal GST tag. The construct was verified by DNA sequencing. The CARM1 (140–480) clone was propagated in *Escherichia coli* DH5α cells. For protein expression, the C41 (DE3) strain of *E. coli* was transformed with the plasmid and plated on LB agar supplemented with ampicillin (100 μg/ml). A 6 L culture was grown in LB medium containing ampicillin (100 μg/ml) and 1% glucose at 37 °C with vigorous shaking (180 rpm) until the absorbance at 600 nm (*A*_600_) reached 0.6. Protein expression was induced by the addition of 0.01 mM IPTG, followed by incubation at 15 °C with shaking (180 rpm) for 24 h. Cells were harvested by centrifugation at 6000 rpm for 15 min at 4 °C and resuspended in ice-cold lysis buffer (50 mM Tris–Cl, pH 8.0, 150 mM NaCl, 5% glycerol, 2 mM β-mercaptoethanol, 0.05% NP-40, and 1 mM PMSF). The cell suspension was lysed by sonication, and the lysate was clarified by ultracentrifugation at 35,000 rpm for 30 min at 4 °C. The GST-tagged protein was affinity-purified using preequilibrated GST beads for 3 h at 4 °C. The beads were washed three times with wash buffer (lysis buffer supplemented with 10 mM ATP and 25 mM MgCl_2_). The GST tag was cleaved by incubation with PreScission protease overnight at 4 °C. The cleaved protein was eluted and further purified by anion exchange chromatography using a Q column (GE HealthCare) with anion exchange buffer (50 mM Tris–Cl, pH 8.0, 150 mM NaCl, and 2 mM β-mercaptoethanol). To generate the CARM1 quadruple mutant (Y153A/E257A/E266A/H414A), site-directed mutagenesis was performed on the WT pGEX-6p-1-CARM1 (140–480) vector using the QuikChange Site-Directed Mutagenesis Kit (Agilent; catalog no.: 200513) according to the manufacturer's protocol. Sequential rounds of mutagenesis were conducted, and the mutations were confirmed by DNA sequencing. Protein concentrations were determined using the Bradford assay, with typical yields of 3 to 4 mg/ml for the WT protein and 3 mg/ml for the mutant.

### Ligand preparation

Metformin hydrochloride (Sigma-Aldrich; catalog no. PHR1084-500MG) was weighed and dissolved in water immediately prior to use. EZM2302 (Cayman Chemical; catalog no. 29954) and TBBD (Cayman Chemical; catalog no. 10569) were dissolved in dimethyl sulfoxide (DMSO; SRL; catalog no. 28580) to prepare stock solutions.

### *In vitro* methyltransferase assay

The methyltransferase activity of purified CARM1 (residues 140–480) was assessed using an *in vitro* assay. CARM1 (1 μM) was incubated with an equimolar concentration of S-adenosyl-L-methionine (SAM; Sigma-Aldrich; catalog no. 20083) for 30 min at 30 °C. Recombinant histone H3 (New England Biolabs; catalog no. M2503) at an equimolar concentration and increasing concentrations of metformin were added simultaneously, and the reaction mixture was incubated for 1 h at 30 °C. The reaction was terminated by placing the samples on ice for 5 min. Protein loading dye was added to the samples, which were then subjected to SDS-PAGE followed by Western blot analysis. The membrane was probed with an anti-histone H3R17me2a antibody (Abcam; catalog no. ab8284) to detect dimethylation at arginine 17 of histone H3. Band intensities were quantified using appropriate imaging software (https://imagej.net/ij/index.html) to assess the effect of metformin on CARM1-mediated methylation.

### Isothermal calorimetry

ITC experiments were carried out using Nano ITC instrument (TA Instruments). Protein and ligand for cell and syringe were prepared in identical buffer solution (10 mM Tris pH 8.0 and 100 mM NaCl) and further dialyzed into the same buffer to minimize heat change due to buffer mismatch. All the ITC experiment was performed using 40 to 50 μM purified CARM1 (140–480) from bacterial expression system in a cell and 1 mM metformin hydrochloride in the syringe. A total of 30 consecutive injections of 2.5 μl of titrant were injected into CARM1 (140–480). Data analysis was done using NanoAnalyze software (TA instruments; tainstruments.com/affinity-itc-auto/).

### Molecular docking

Molecular docking of metformin with CARM1 was performed using the crystal structure of CARM1 (PDB ID: 4IKP) retrieved from the RCSB PDB. The protein structure was prepared using PyMOL (Schrödinger, LLC; https://www.pymol.org) and AutoDockTools by removing water molecules and adding hydrogen atoms. The three-dimensional structure of metformin was obtained from PubChem and prepared using Open Babel (https://openbable.github.io), which included hydrogen addition and geometry optimization. Docking simulations were carried out using DiffDock, a machine learning-based docking tool, which generated multiple binding poses. The pose with the highest predicted binding affinity was selected for further analysis. Key interactions between metformin and the active site residues of CARM1 were identified and visualized using PyMOL (Schrödinger, LLC; The PyMOL Molecular Graphics System, Version 2.0).

### Cell culture

HepG2 cells were cultured and maintained in Dulbecco's modified Eagle's medium (DMEM; Gibco; catalog no. 12800-017) supplemented with 10% fetal bovine serum (Gibco; catalog no. 16140071), 1% antibiotic–antimycotic (Gibco; catalog no. 15240062), and 1% nonessential amino acids (Gibco; catalog no. 11140050). Cells were incubated at 37 °C in a humidified atmosphere containing 5% CO_2_. For protein extraction, HepG2 cell pellets were resuspended in 2× Laemmli buffer (125 mM Tris–Cl [pH 6.8], 4% SDS, 20% glycerol, 0.004% bromophenol blue, and 10% 2-mercaptoethanol), boiled at 100 °C for 5 min, and vortexed three times to ensure complete lysis. Total protein concentration was quantified using the Bradford assay. For transfection, Lipofectamine 2000 reagent (Invitrogen; catalog no. 11668019) was used according to the manufacturer's protocol. Briefly, HepG2 cells were allowed to reach 70 to 80% confluency, after which the media was replaced with Opti-MEM (Gibco; catalog no. 31985070). Lipofectamine 2000 and DNA were mixed in serum-free Opti-MEM at a ratio of 1:2 (v/v) and incubated for 20 min at room temperature to form complexes. The mixture was added dropwise to the cells, and overexpression was allowed to proceed for 24 h. Metformin hydrochloride was prepared in sterile water and added to the cell culture media at the desired concentration. TBBD and EZM2302 were dissolved in DMSO and added to the cells, ensuring that the final concentration of DMSO did not exceed 10% (v/v). Cells were treated with metformin for 12 h, while EZM2302 and TBBD treatments were carried out for 48 h. Following treatment, cells were harvested, rinsed twice with cold PBS, and processed for downstream experiments.

### Western blot analysis

Lysate samples were resolved by SDS-PAGE and transferred onto a nitrocellulose membrane (Millipore; catalog no. HATF00010). The membrane was blocked with 5% bovine serum albumin (SRL; catalog no. 83803) in 1× Tris-buffered saline containing 0.1% Tween-20 (TBST) for 1 h at room temperature. Following blocking, the membrane was incubated with primary antibodies specific to the target proteins at 4 °C overnight with constant shaking. After incubation, the membrane was washed three times with TBST for 5 min each. The membrane was then probed with horseradish peroxidase-conjugated secondary antibodies, either goat anti-rabbit IgG (Invitrogen; catalog no. 31460) or goat anti-mouse IgG (Invitrogen; catalog no. 62-6520), for 3 h at room temperature. After washing the membrane three times with TBST for 5 min each, protein bands were visualized using an enhanced chemiluminescent substrate (Thermo Fisher Scientific; catalog no. 24580). The chemiluminescent signal was detected and imaged using the iBright FL1500 imaging system (Thermo Fisher Scientific).

### Quantitative real-time PCR and ChIP

Total RNA was isolated from cells using TRIzol reagent (Invitrogen; catalog no. 15596026) following the manufacturer's instructions. Complementary DNA was synthesized from 1 μg of total RNA using the Verso cDNA Synthesis Kit (Thermo Fisher Scientific; catalog no. AB1453A). Quantitative RT-PCR was performed using SYBR Green Mastermix (Bio-Rad; catalog no. 1725121) in a Bio-Rad CFX96 Real-Time PCR system. Reactions were carried out in a final volume of 10 μl, with cycling conditions as follows: 94 °C for 15 s, annealing at the primer-specific temperature for 30 s, and extension at 72 °C for 30 s. Annealing temperatures were optimized based on the melting temperature (Tm) of the primers. Each reaction was performed in triplicate to ensure reproducibility. ChIP assays were performed using HepG2 cells as previously described (60). Briefly, cells were cross-linked with 1% formaldehyde, and chromatin was sheared by sonication to obtain DNA fragments of 200 to 500 bp. Immunoprecipitation was carried out using specific antibodies against H3R17me2a (Abcam; catalog no. ab8384) and CARM1 (Cell Signaling Technology; catalog no. 3379). Normal sheep serum IgG (Rockland; catalog no. B311) was used as a negative control. After immunoprecipitation, protein-DNA complexes were eluted, and cross-links were reversed. DNA was purified and analyzed by quantitative real-time PCR using primers specific to the target genes. All ChIP experiments were performed in three independent biological replicates, with three technical replicates.

### Animal experiments

Protocols for animal experiments were approved by Institutional Animal Ethics Committee at CSIR-IICB under the aegis of Committee for Control and Supervision of Experiments on Animals (CPCSEA), Ministry of Environment and Forest, Government of India. WT 6–8-week-old C57BL/6 male mice were housed at individually ventilated cages. All mice used in this study were maintained at 22 ± 1 °C in a 12 h light/dark cycle at with ad libitum food and water, in a specific pathogen-free facility. Studies were conducted during the light cycle. Mice were fed with metformin, administered *via* oral gavage at a dosage of 350 mg/kg body weight, daily, for 1 week (n = 5 for control group and n = 7 for metformin fed). Feeding time was fixed and interval between each feeding time was 24 h. Mice were fasted for 4 h prior to sacrifice. Mice livers were cut into small sections and stored in liquid nitrogen/TRIzol for downstream experiments.

### Study approval

All experiments were done according to the Institutional Animal Ethics Committee guidelines of CSIR-Indian Institute of Chemical Biology and approved by the Committee for Control and Supervision of Experiments on Animals (CPCSEA), Ministry of Environment and Forest, and Government of India.

### Mouse primary hepatocyte culture and treatment

Primary hepatocytes were isolated from 6–8-week-old C57BL/6 male mice housed under a 12 h light/dark cycle at 22 ± 1 °C with free access to food and water. The liver was perfused with Hanks balanced salt solution I (0.4 mM KH_2_PO_4_, 5 mM KCl, 4 mM NaHCO_3_, 140 mM NaCl, 0.5 mM MgCl_2_·6H_2_O, 0.3 mM Na_2_HPO_4_, 0.4 mM MgSO_4_.7H_2_O, 6 mM glucose, 25 μM Hepes, and 0.5 mM EDTA; pH 7.4) followed by digestion with prewarmed Collagenase D (Roche; catalog no. 11088858001). The isolated hepatocytes were cultured in William's E Medium supplemented with 1% penicillin–streptomycin solution (HiMedia; catalog no. A001A) and 5% fetal bovine serum (Gibco; catalog no. 16140071). Cells were plated on collagen-1, rat tail precoated plates (Gibco; catalog no. A1142801) and maintained in Hepatocyte Culture Medium containing HBM Basal Medium with supplements (Lonza; catalog no. 11695450). Metformin hydrochloride (Sigma-Aldrich; catalog no. PHR1084-500MG) was prepared in sterile water and administered to the cultured hepatocytes at a final concentration of 10 mM for 12 h. After treatment, cells were harvested and lysed for protein extraction. Western blot analysis was performed as described previously to assess the effects of metformin on target proteins.

### Immunohistochemistry

The control and metformin-treated mice liver tissues were stored in 10% neutral buffer formalin and after dehydration, paraffin blocks were prepared. The 3 μm paraffin section was embedded in a poly-L-lysin-coated slide. The deparaffinization was done by heating at 65 °C for 60 min followed by washing in xylene, 100% ethanol, 90% ethanol, 80% ethanol, and 70% ethanol, respectively for 5 min each and then kept it in water for 10 min. The antigen retrieval was done by boiling in Tris–EDTA pH 9.0 buffer in a microwave oven for 12 min. After complete cooling, the immunohistochemical staining with anti-H3R17me2a antibody was performed by using an IHC kit (Abcam, ab64264) following the manufacturer's protocol. In brief, the tissues were blocked with peroxide blocker, and protein blocker followed by primary antibody binding for 60 min at room temperature and secondary antibody binding for 15 min. The tissues were stained with 3,3′-diaminobenzidine and imaged with a bright field light microscope in 20× objective lens. The quantification was done by using ImageJ.

### Extracellular flux analysis

The ECAR was measured using an extracellular flux analyzer (Seahorse XFe24, Agilent Technologies) following the manufacturer's protocol (47). HepG2 cells were seeded in a 24-well Seahorse XF cell culture microplate and cultured for 24 h in complete DMEM media at 37 °C in a 5% CO_2_ incubator. After 24 h, CARM1 overexpression and metformin treatment were performed as described. Prior to the assay, cells were washed three times with ECAR assay media (XF Base Medium Minimal DMEM; Agilent; catalog no. 102353-100), supplemented with 1 mM sodium pyruvate and 2 mM GlutaMAX (Gibco; catalog no. 35050061), and adjusted to pH 7.4. After washing, 450 μl of ECAR assay media was added to each well, and the plate was incubated in a non-CO_2_ 37 °C incubator for 1 h to allow temperature and pH equilibration. The Seahorse XF sensor cartridge was hydrated with XF calibrant (pH 7.4; Agilent; catalog no. 100840-000) for 24 h at 37 °C in a non-CO_2_ incubator. ECAR inhibitors, including 10 mM glucose, 1.5 μM oligomycin A, and 15 μM 2-deoxy-D-glucose, were loaded into their respective ports in the cartridge. The cartridge was then calibrated according to the manufacturer's instructions.

After calibration, the cell culture plate was loaded into the Seahorse XFe24 analyzer, and the ECAR assay was performed. Data were collected and analyzed using Wave Desktop 2.6.3 software (https://www.agilent.com/en/products/cell-analysis/software-download-for-wave-desktop) (Agilent Technologies). Further statistical analysis and graphical representation were performed using GraphPad Prism 8.4.2 (https://www.graphpad.com/features).

### Glucose production assay

HepG2 cells were seeded in 12 well dishes in 25 mM glucose DMEM media. After CARM1 overexpression and subsequent metformin treatment, the 25 mM glucose DMEM media was replaced with no glucose, no phenol red containing DMEM media supplemented with 20 mM sodium lactate and 2 mM sodium pyruvate. After 7 h of incubation, 10 mM cAMP (Sigma-Aldrich, cat no-A6885) and 5 μM dexamethasone (Sigma-Aldrich, cat no-D4902) were added to the media and incubated for 9 h (48). Then, the media were collected, and the amount of glucose was measured using a Glucose (GO) assay kit (Sigma-Aldrich, cat no-GAGO20) according to the manufacturer protocol using a 96-well plate reader and normalized with protein concentration.

### Statistical analysis

Unpaired two-tailed Student's *t* test and one-way ANOVA were performed to determine the significant differences between the groups for quantitative RT-PCR and ChIP, respectively. Data were collected from experiments performed in triplicate (n = 3). Error bars indicate the standard deviation of the mean for the technical replicates (*i.e.*, mean ± SEM). The statistical significance was considered as (ns) *p* 0.05, ∗*p* ≤ 0.05, ∗∗*p* ≤ 0.01, ∗∗∗*p* ≤ 0.001, and ∗∗∗∗*p* ≤ 0.0001. All statistical analyses were performed using GraphPad Prism 8.0 (GraphPad).

## Data availability

All data in this manuscript are contained within the manuscript.

## Conflict of interest

The aupthors declare that they have no conflicts of interest with the contents of this article.
